# Low Complexity System on Chip Design to Acquire Signals from MOS Gas Sensor Applications

**DOI:** 10.3390/s21196552

**Published:** 2021-09-30

**Authors:** Juan B. Talens, Jose Pelegri-Sebastia, Maria Jose Canet

**Affiliations:** 1 IGIC Institute, Campus Gandia, Universitat Politècnica de València, 46730 Gandia, Spain; juatafe@upv.es; 2 Instituto de Telecomunicaciones y Aplicaciones Multimedia, Campus Gandia, Universitat Politècnica de València, 46022 Valencia, Spain; macasu@eln.upv.es

**Keywords:** sigma delta, ADC, SoC, Altera, DE-1-SOC, MOS gas sensor, LVDS, FPGA

## Abstract

Analog signals from gas sensors are used to recognize all types of VOC (Volatile Organic Compound) substances, such as toxic gases, tobacco or ethanol. The processes to recognize these substances include acquisition, treatment and machine learning for classification, which can all be efficiently implemented on a Field Programmable Gate Array (FPGA) aided by Low-Voltage Differential Signaling (LVDS). This article proposes a low-cost 11-bit effective number of bits (ENOB) sigma-delta Analog to Digital Converter (ADC), with an SNR of 75.97 dB and an SFDR of 72.28 dB, whose output is presented on screen in real time, thanks to the use of a Linux System on Chip (SoC) system that enables parallelism, high-level programming and provides a working environment for the scientific treatment of gas sensor signals. The high frequency achieved by the implemented ADC allows for multiplexing the capture of several analog signals with an optimal resolution. Additionally, several ADCs can be implemented in the same FPGA so several analog signals can be digitalized in parallel.

## 1. Introduction

In order to understand the metal oxide semiconductors (MOS) which are used as gas sensors in multiple applications [1,2,3,4], we must consider them at the molecular level. Volatile organic compounds such as methane can react with oxygen, yielding carbon dioxide and water as products (Equation (1)):

CH_4_ + 2O_2_ →CO_2_ + 2H_2_O (1)

**Figure 1:**
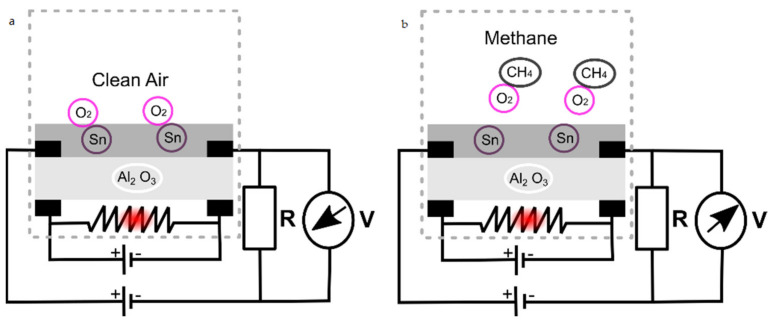
(**a**) Sensor reaction with clean air. (**b**) Sensor reaction with methane.

MOS sensors mainly use tin dioxide, indium oxide or tungsten oxide as semiconductors and alumina as substrate [5,6]. The use of a catalyst reduces the amount of heat necessary to provoke the reaction. Figure 1 shows the sensor reaction in two cases: clear air and in reaction with methane. The dashed square is the gas sensor, which includes four terminals, a heater and Al_2_O_3_ as substrate. In clean air (Figure 1a), a tin oxide semiconductor has high resistivity which prevents electron movement. When methane gas is introduced (Figure 1b), the reaction seen in Equation (1) occurs. As a result of this movement of electrons, the resistivity of the gas sensor decreases and allows current flow in the sensor. This type of sensor, which has a resistive output, is simple to use and inexpensive [4,6]. When connecting a resistor R as shown in Figure 1a, a voltage divider is formed, and the output voltage can be measured. That is, the gas sensor becomes an analog-output sensor.

Due to the nature of the reaction and the polarity of the current, the measured voltage is always positive. On the other hand, it presents some thermic noise and an offset voltage due to the heater provided and the material’s conductivity.

**Figure 2:**
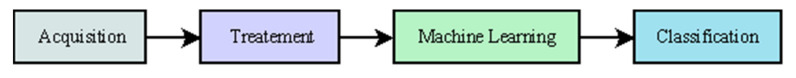
Stages in an e-Nose classifier.

As some substances such as urine or feces have VOCs [7,8,9,10], gas sensors are an excellent option to obtain information from them without contact. Many electronic noses (e-Noses) have been proposed in the literature [11,12,13,14,15] to analyze data from gas sensors. The first stage of an e-Nose consists of an array of gas sensors connected to an ADC, that is, the acquisition stage, which is the focus of this paper. Figure 2 shows the usual stages in an e-Nose that classifies substances. After the acquisition, the digital signal can be processed (filter, windowing, normalization, offset compensation) in the same e-Nose or in a PC. Lastly, machine learning and classification stages are usually neuronal networks [16,17] in charge of training the system and recognizing the sample from gas sensor data [18], respectively.

In this paper, a solution for the acquisition stage that can be implemented by taking advantage of the LDVS input of an FPGA with a Linux System on Chip (SoC) system is proposed, which allows the implementation of the complete e-Nose Classifier in Figure 2 in the same device. Thus, our proposal is an alternative to microprocessor boards such as Arduino’s and ADC-specific boards, which cannot be used for complex machine learning and require a PC. Our proposal also has the advantage of including an operating system, which allows us to develop other interesting applications such as the capture visualization. 

Regarding the implementation of the acquisition stage using LDVS inputs of an FPGA, several proposals can be found in the literature. In [19], an 8-bit Approximation Register (SAR) and a 10-bit sigma delta are proposed, which require 135 and 1 k Lookup Tables (LUTS), respectively. In both approaches, an RC circuit connects the 1-bit DAC output to the FPGA LVDS input. The first solution proposes a 4-bit SAR, but only the most significant bit is connected to the RC. This is a low-cost solution for low-frequency analog input signals (up to 1 kHz). The second solution in [19] consists of a sigma-delta modulator with a sampling register and a cascade integrated comb (CIC) filter. This is more complex, but it can be used for analog input signals of up to 50 kHz. In [20], a 12-bit sigma-delta ADC similar to the one proposed in [19] is presented. The main difference between both proposals is that [20] uses an equalizer and a finite impulse response (FIR) filter apart from the CIC that adds more complexity to the design (700 LE-Logic Elements are required).

The purpose of this paper is to design an ADC for analog e-Nose signals. The main characteristic of these signals is their low frequency, so we do not need complex ADC techniques that include a CIC or an equalizer. The ADC proposed in the paper is based on the sigma-delta technique, but the CIC filters in [19,20] are substituted by a registered accumulator. The proposed solution is valid for low-frequency analog signals and allows a reduction in the complexity of the ADC.

Apart from the implementation of the ADC on FPGA, the scope of this paper also covers the hardware configuration of the SoC and the software programming that allows one to view the output of the gas sensor in a video graphics array (VGA) monitor. For the application under study, as many ADCs as gas sensors in the e-Nose need to be implemented, which can be achieved by implementing several ADCs in the same FPGA and/or multiplexing the gas sensors’ outputs.

This paper is organized as follows: the ADC’s specifications, the evaluation board used, and the test of the designed ADC are detailed in Section 2; Section 3 is dedicated to the implementation (hardware and software); Section 4 describes the results and conclusions are derived in Section 5.

## 2. Specifications, Materials and Test

This section presents the specifications of the input signal, which is obtained from an e-Nose, and the materials used to test the implemented sigma-delta ADC, including the board DE1-SoC from Terasic [21].

### 2.1. Input Signal

**Figure 3:**
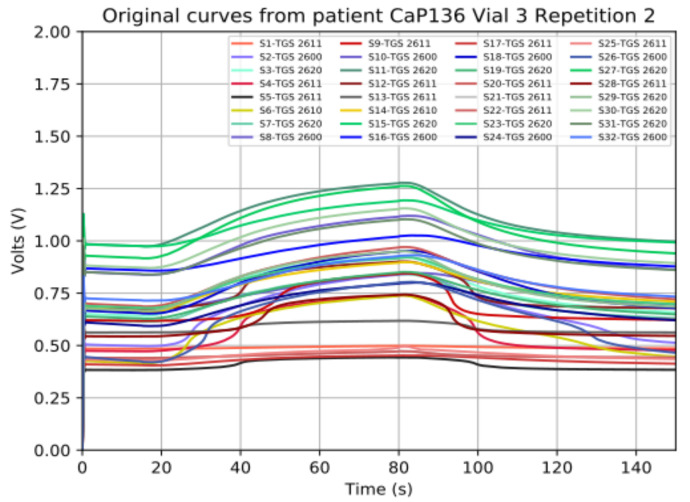
Curves of a prostate cancer patient extracted with the e-Nose of 32-sensor MOOSY32 after the ADC conversion.

**Figure 4:**
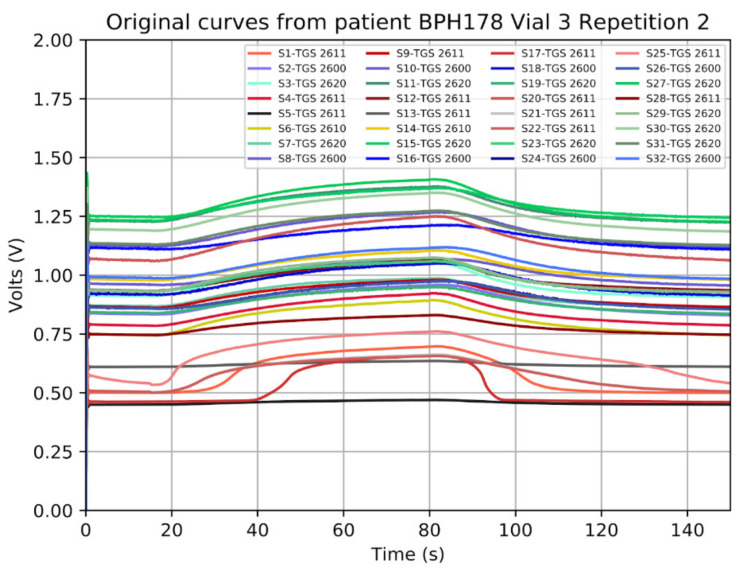
Curves of benign hyperplasia patients extracted with the e-Nose of 32-sensor MOOSY32 after the ADC conversion.

As commented above, the analog signal to acquire is obtained from an e-Nose. In [22], an e-Nose named MOOSY32, which is composed of 32 sensors, is used to identify an olfactory pattern of prostate cancer in urine. This e-Nose uses a sample rate of 100 Hz, 14-bit and 12-bit ADC National Instruments boards. Figure 3 and Figure 4 show the captured signals, truncated to two decimal places, that were used to identify patterns. As can be seen, the signals change slowly (the steepest slope found corresponds to a frequency of 15 mHz) and they are always positive, lower than 2 V and with some offset.

In this paper, we propose substituting the ADC National Instruments boards with a sigma-delta ADC implemented on an FPGA, where post-processing could also be implemented in the future. As the signal only requires two decimal places after conversion, our target is to obtain an ADC with a minimum of 9 bits of resolution.

### 2.2. Board DE1-SoC from Terasic

The proposed sigma-delta ADC for MOS gas sensor signals was implemented on a Terasic DE1-SoC [21], which is a development kit DE1-SoC. This card is a development kit around a system on chip (SoC) that combines a Cyclone V FPGA with an ARM cortex-A9 (HPS4). This FPGA contains 85 k programmable elements. Table 1 details the FPGA included in the DE1-SOC card.

### 2.3. Test

#### 2.3.1. Initial Test Using Simulink

**Figure 5:**
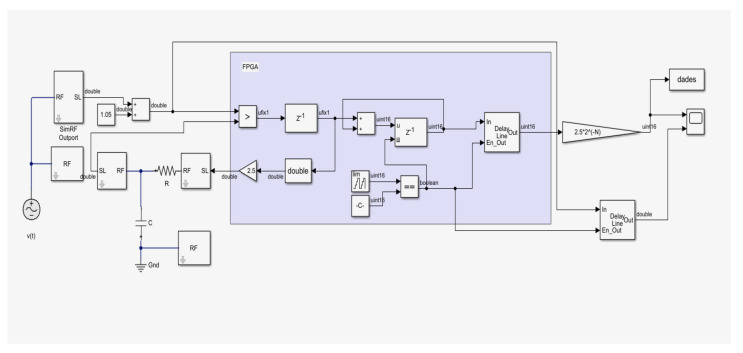
Test model Σ∆ implemented in Simulink.

First, a simulink model of the sigma-delta ADC was created to validate the design. Then, after implementing the ADC on the FPGA, a final test was performed. Figure 5 shows the simulink model of the designed ADC that was used to simulate its behavior before starting the implementation on FPGA.

#### 2.3.2. Final Test

**Figure 6:**
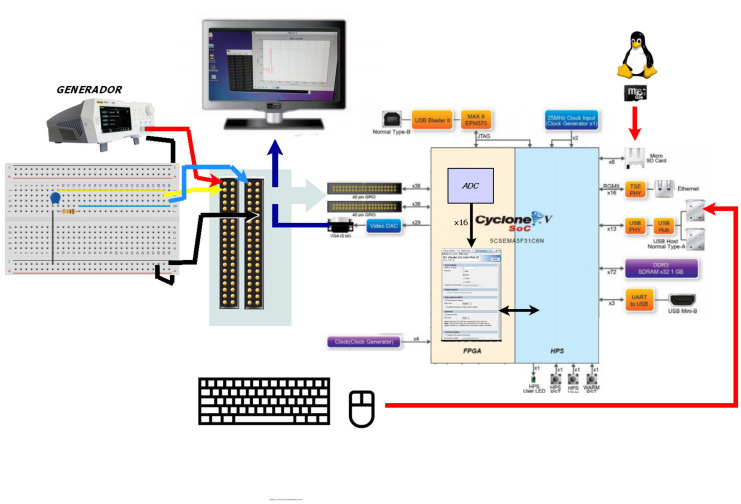
Test of the ADC converter.

Once the desired results were obtained with Simulink, the sigma-delta ADC converter was implemented on FPGA (see Section 3) and then tested using the scheme shown in Figure 6. The analog input signal was obtained from a generator Rigol DS1054, configured to generate a low-frequency sine signal. The ADC was implemented on the FPGA, but it also required an external RC circuit. This RC circuit and the signal generator were connected to the GPIO pins, whereas a monitor, a mouse and a keyboard were connected to their corresponding ports of the development board. In this way, the use of an external PC to perform the test and to process the output of the ADC was avoided. The implemented ADC was managed by the operating system (HPS-hard processor system), which also controlled an SD card with the Linux system, the keyboard and the mouse. The digital output of the ADC was represented on the screen and stored to be processed. In addition to the ADC component, the VGA display drivers were also implemented on the Cyclone V FPGA. In this way, the signal generated is captured and showed in real time.

## 3. Implementation 

The system developed consists of a few elements mapped as hardware on the FPGA and an app to control that hardware. This section details the designed low-complexity system on chip design for the signal acquisition of MOS gas sensor applications, which includes: (a) the ADC; (b) the mapping of this ADC in an Altera Intellectual Property (IP), which is necessary for the HPS to control the ADC; (c) the application to visualize the captured signal on the monitor. The implemented ADC requires only 172 ALMs (Adaptive Logic Modules) on the FPGA.

### 3.1. ADC

**Figure 7:**
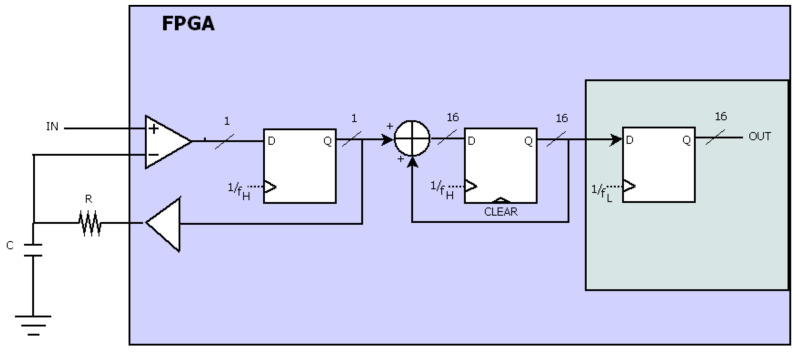
Theoretical model Σ∆ implemented.

As said above, the proposed ADC is based on the sigma-delta technique, but this is simplified thanks to fact that signals captured by e-Noses have very low frequencies. The block diagram of our proposal can be seen in Figure 7. Five stages can be distinguished: an RC filter, a comparator (implemented as LVDS), a 1-bit DAC, an accumulator and a sample and hold (S&H) register. All stages work at a high frequency, (f_H_) but the S&H register works at a low frequency (f_L_), that is, the output sample frequency, f_s_ = f_L_. To guarantee a resolution of 9 bits, 16 bits are used internally for the accumulator and S&H register. For this reason, f_H_ was set to f_H_ = f_L_·2^16^. The accumulator can grow from 0 to 65,535; if the input signal is greater than the registered input, then the output of the accumulator was increased by one; if not, it remained the same. In this way, the register of the accumulator stores the value of the input signal after 65,536 cycles. Then, the S&H captures this value at f_L_ and the register of the accumulator is cleared (every 65,536 cycles) to start to process the following sample of the analog input.

#### 3.1.1. Filter RC

**Figure 8:**
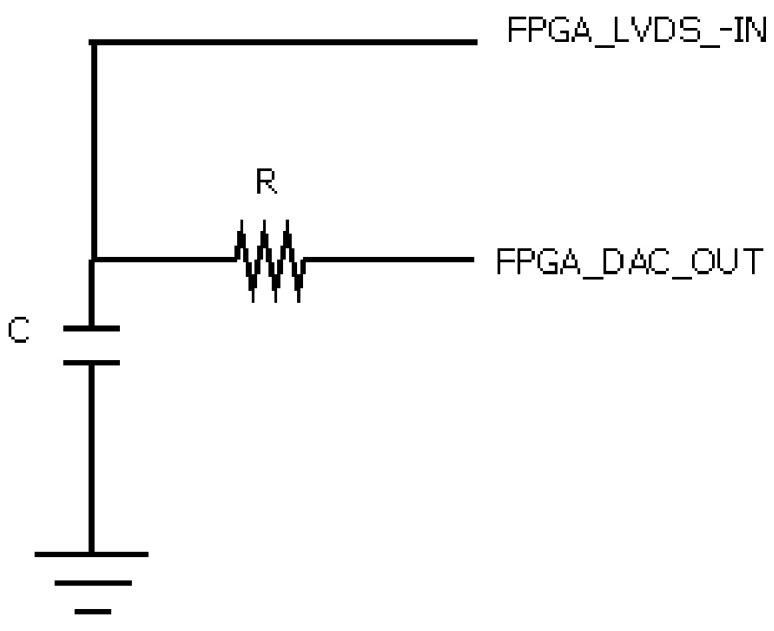
Stage filter RC.

**Figure 9:**
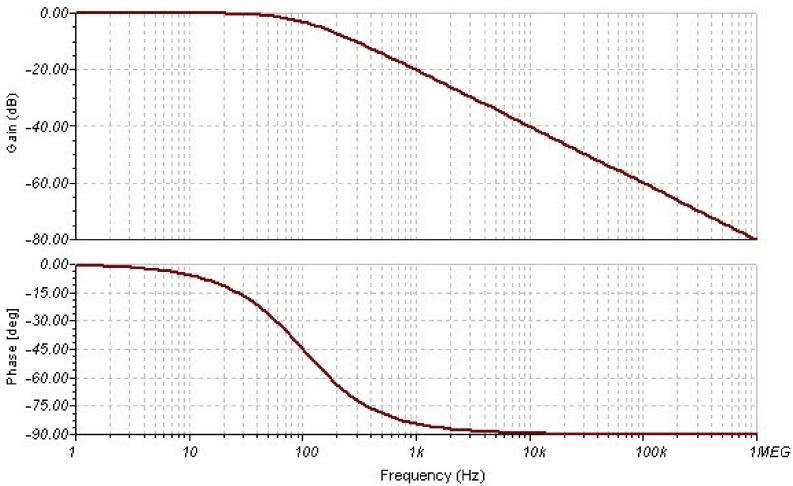
Filter response pole in 100 Hz, R = 10.61 kΩ, C = 150 nF.

The RC filter (Figure 8) integrates the output of the 1-bit DAC. The output of the RC filter was compared with the analog input (IN) using a LDVS input of the FPGA. The values of R and C were obtained so that a pole is provided at f_L_ = f_S_. Figure 9 shows the frequency response of the filter for fs = 100 Hz.

#### 3.1.2. LVDS

**Figure 10:**
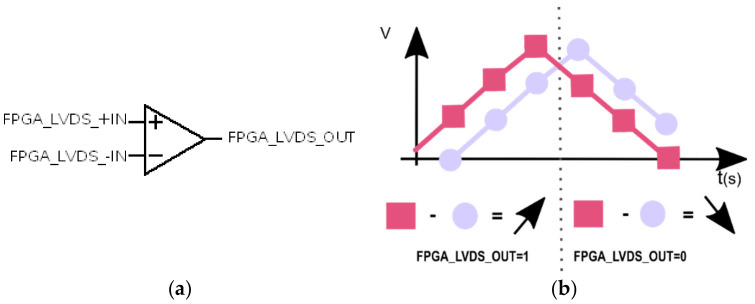
(**a**) Scheme LVDS and (**b**) stage LVDS.

LVDS input is similar to an operational amplifier; it has an inverting input, a non-inverting input, and an output, as can be seen in Figure 10a. It works non-linearly, internally subtracting both inputs [9] and giving FPGA_LVDS_OUT = 1 if the result is positive and FPGA_LVDS_OUT = 0 if the result is negative. They are manufactured with a certain hysteresis because their purpose is to offer a stable output with small variations in voltage. This produces a dismount of its resolution [12]. The threshold or minimum differential input voltage required by the component for a common mode voltage of 1.25 V is 100 mV [17].

Figure 10b represents the inputs of the LVDS, the analog input from the sensor (in squares) and the output of the RC filter (in circles), which comes from the 1-bit DAC that works at f_H_. When the slope of the analog input is positive, the output of the LVDS will be 1; in any other case, it will be 0. The LVDS input cannot detect positive slopes lower than its minimum differential input voltage. When this happens, the 1-bit DAC output will be 0 and the voltage in the LVDS non-inverting input will decrease thanks to the integration made by the RC filter, so the differential input voltage increases. If the differential input voltage is higher than the minimum, the LVDS will be 1 (the positive slope is detected); if not, the 1-bit DAC output will be 0 and the LVDS non-inverting input will decrease again. In this way, the differential input voltage is continuously increased until the positive slope is detected.

#### 3.1.3. 1-bit DAC

**Figure 11:**
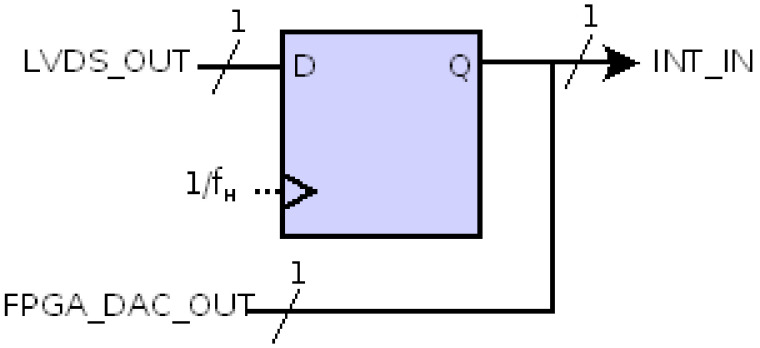
Stage 1-bit DAC.

It is necessary to obtain a delayed copy of the input signal to be digitized. Figure 11 shows the 1-bit digital-to-analog converter consisting of a 1-bit register at frequency f_H_. Its output is connected to an FPGA output buffer to be integrated by the RC filter, and also to the integrator. In our e-Nose application, we need fs = f_L_ = 100 Hz, so f_H_ should be 100 × 2^16^ = 6,553,600 Hz. Thus, 65,536 digital-to-analog conversions are required to obtain an output sample at f_L_.

**Figure 12:**
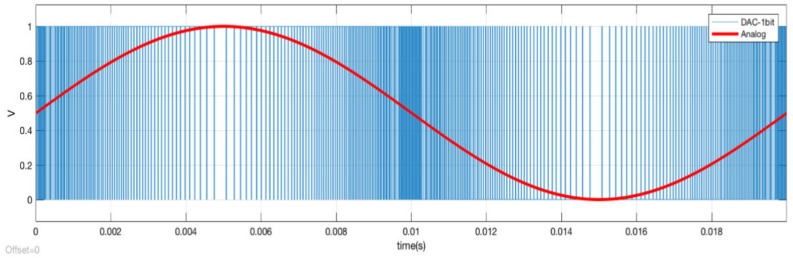
Sinusoidal signal input analog v(t) = 0.5 + 0.5 sin (2π50t) output.

Figure 12 shows the output of the 1-bit DAC (INT_IN in Figure 10b) when the analog signal (IN in Figure 6) is sinusoidal (amplitude = 0.5 V, offset = 0.5 V and frequency = 50 Hz). To see a representative output, f_H_ was set to 65,536 Hz. Note the frequency modulation of the 1-bit DAC output, which follows the slope of the analog input signal.

#### 3.1.4. Input/Output Intel FPGA Intellectual Property

**Figure 13:**
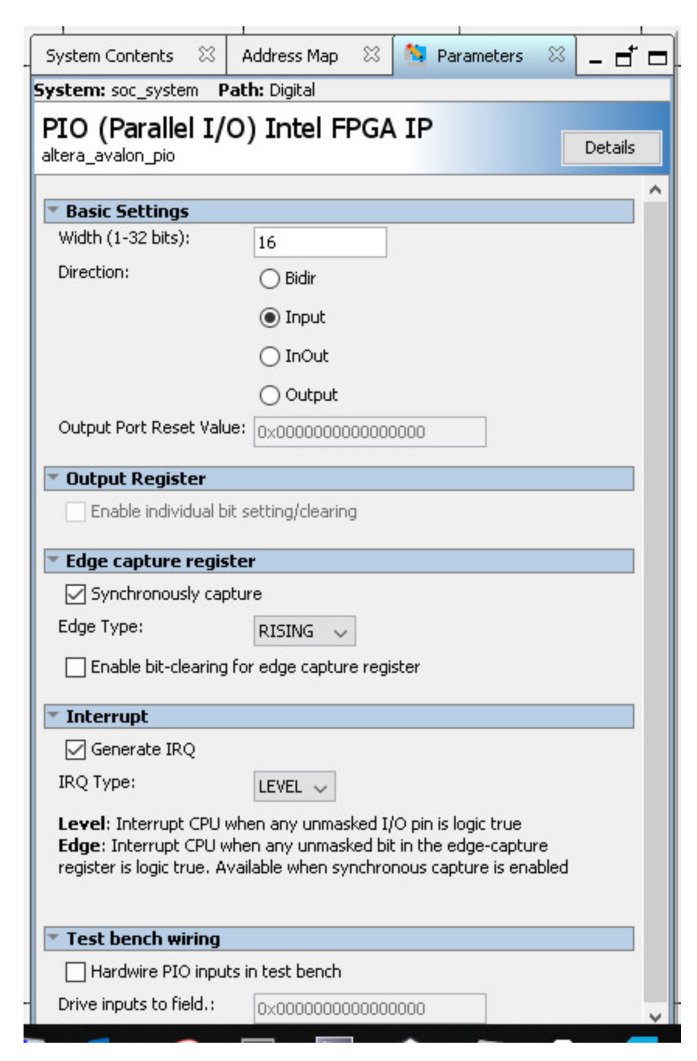
Input component altera_avalon_IO with 16 bits.

The implemented ADC is integrated as an Altera Intellectual Property (IP) and connected to bus avalon with altera_avalon_pio component. In this way, the ADC is connected to the HPS (Hard Processor System) as can be seen in Figure 6. Figure 13 shows how it is configured. This component is an input because it collects data from the ADC converter output to be read by the operating system. This is possible thanks to the memory mapping of the component within the system bus.

### 3.2. Application to Visualize the Captured Signal

In addition to the design of the ADC, we also designed an application to store and represent the captured signal on-screen. To achieve this, the ARM needs to use the Linux operating system (Ubuntu, London, UK) to control the resources of the development board, including the FPGA. Next, the configuration of the board and the design of the program used to obtain this representation are explained.

#### 3.2.1. Configuration of Board DE1-SoC to Use SoC

First, the file system on an SD card needs to be prepared with the file system and the system image, and it should include:

- A Pre-loader or secondary U-boot loading program generated with bsp-editor tool. The U-boot loads three files from the first FAT partition of the SD.
- The Device Tree Blob or device tree, which tells the Kernel the hardware that is connected. Some details need to be input manually by editing the tcl files and removing duplicates from the dts file before generating the dtb file.
- The Raw Binary File or bit stream to configure the FPGA system. This file is generated by the convert programming files tool (Quartus).
- Linux kernel or system kernel downloaded from ©Terasic.
- Linux Root File system or file system and folders to mount the Linux. Fortunately, the ©Terasic website offers an image of the SD with a system of examples that can be used as a starting point.

On the other hand, the following components need to be created using the Platform Designer tool:

- FPGA Clock Signal from a board of 50 MHz.
- Clock signal from a pll component (f_H_).
- ADC component designed in this paper.
- Additional input component to connect the ADC component with the bus.
- HPS component, interrupt capturer component and VGA controller component, which can all be obtained from the example given by ©Terasic.

Finally, the IP catalogue tool is used to generate IPs for the LVDS inputs and for the buffer output; input pins are configured as LVDS and output pins as buffers. 

#### 3.2.2. Program Design

**Figure 14:**
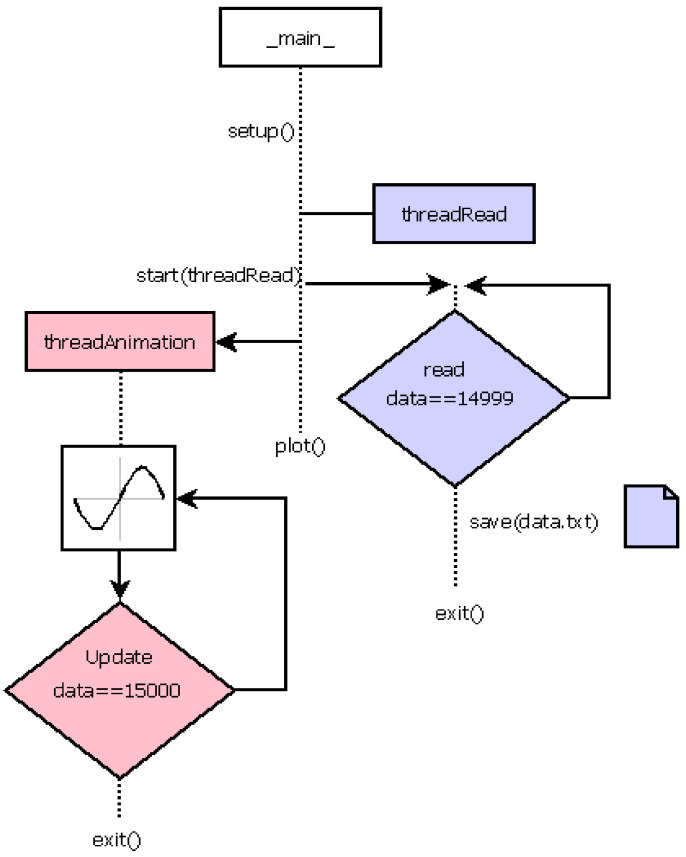
Python3 script flowchart.

The HPS on the evaluation board has two cores, and each core has one thread, but it has to complete more than two tasks: be aware of the operative system interruptions, read data from the ADC and plot these data on-screen in real time. The data from the ADC change every 10 ms, so the data-reading task needs to have priority over the rest of the tasks. Figure 14 shows the flowchart of the developed script, which contains two threads: one to read the data, which uses a token to block the CPU while it is reading and otherwise puts this thread to ‘sleep’, and another for the representation, called Animation, which can be executed while the reading thread is ‘sleeping’. Once the last sample of the ADC is read, all the captured data are written to a file.

An alternative to our proposal is to use both cores in parallel. In this case, two independent processes are required (reading and representation), but both require the same input data (the ADC output). As processes do not include data or memory space, a pipe needs to be used, so more time is required to run a process and put the other process ‘on hold’ when a core needs to attend to other processes in the system. On the contrary, the change of execution thread in our proposal, which uses two threads in the same process, is faster due to the fact that threads share data and memory space.

## 4. Results

This section presents the results obtained from the simulation and from the implementation of the proposed ADC.

### 4.1. Simulation Results

**Figure 15:**
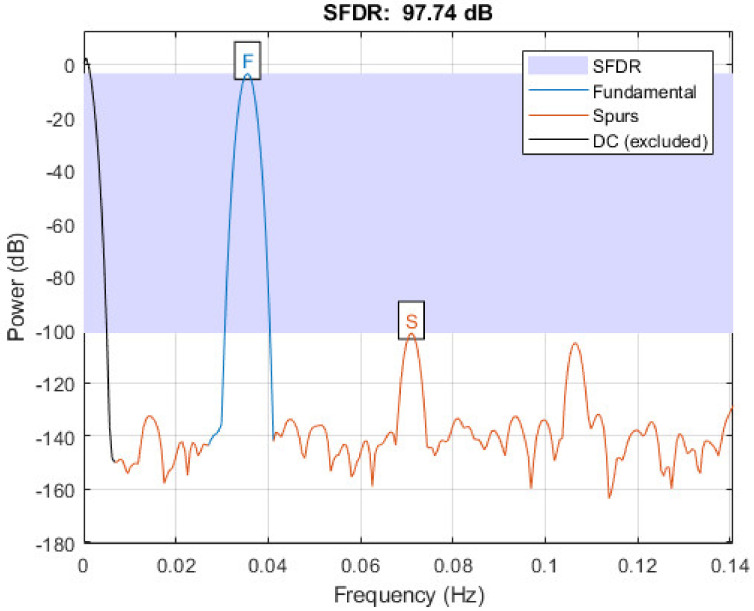
SFDR from 35.5 mHz Matlab signal simulation.

First, we obtained the effective number of bits (ENOB) of the ADC Simulink model for a 16-bit sine input signal. Table 2 shows the parameters of the ADC converter (fL, fH, R and C) and the resulting ENOB, 14.75 dB, which is obtained from the SFDR (Spurious Free Dynamic Range) measure in Figure 15 using Equation (2), where N is the number of bits.

SFDR = 6.02N − 3.92 dB.(2)

Note that an ideal comparator is used in this model, so the non-linearity of the LDVS is not affecting the measure.

### 4.2. Implementation Results

In this section, the results of the test of the ADC implemented on Terasic DE1-SoC are detailed. To obtain these results, we used the scheme showed in Figure 6, with a sine input signal of 35.5 mHz, a maximum at 2 V and a minimum at 100 mV.

#### 4.2.1. Gain and Offset

Next, the output of the implemented ADC needs to be adjusted to obtain a signal with the same amplitude and offset as the sine input. In this way, we are compensating for the non-linearities introduced by the LVDS input.

**Figure 16:**
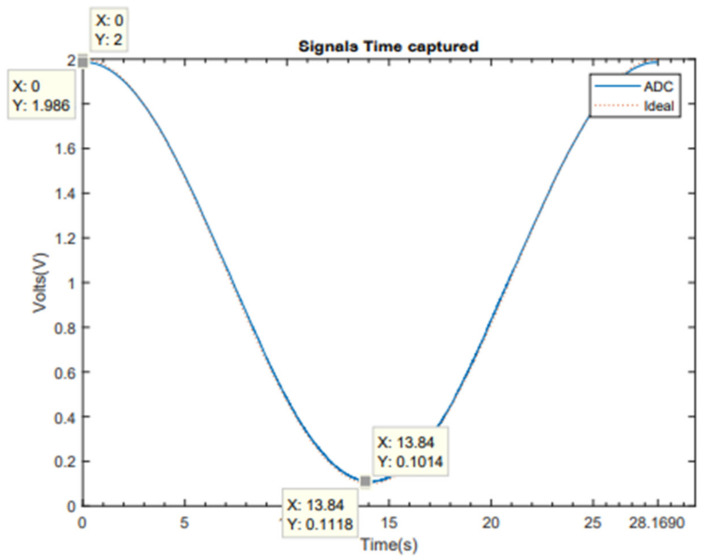
Ideal signal with captured signal.

Figure 16 compares the ADC’s output with the ideal sine signal. A slight difference in gain and offset can be observed, which is compensated by the software (multiplying by 0.1014/0.1118 = 0.907).

**Figure 17:**
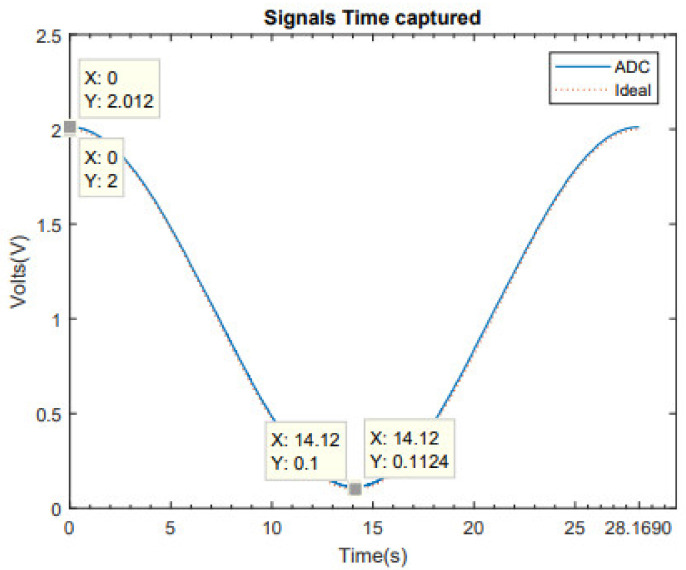
Ideal signal with captured signal with gain adjustment.

Figure 17 shows the ADC’s output once the gain is compensated, so the difference in the offset is calculated by 2.012 − 2 = 12 mV, 0.1124 − 0.1 = 12 mV. This offset is also compensated by the software, so we obtain a sine signal with the same amplitude and offset as the input.

#### 4.2.2. Effective Number of Bits (ENOB and SFDR)

The effective number of bits (ENOB) for a sine input signal is obtained with (3):

ENOB = (SNR_db_ − 1.761)/6.02 (3)

**Figure 18:**
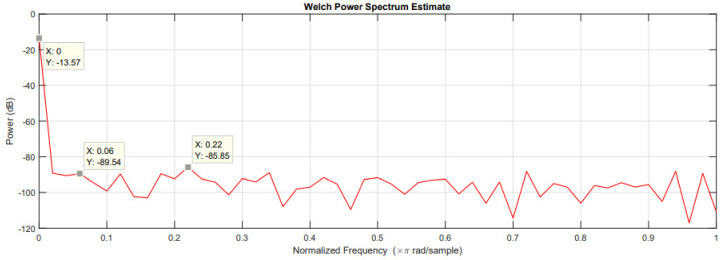
Power spectrum estimation for the ENOB calculation.

**Figure 19:**
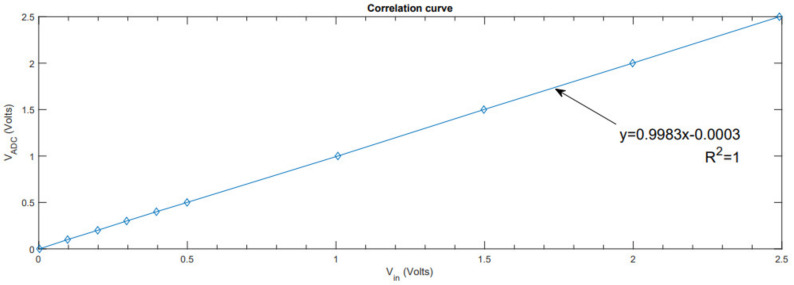
Transfer function with captured signal.

Figure 18 shows the power spectral analysis of the captured signal. The measured signal-to-noise ratio (SNR) is 75.97 dB, so the effective number of bits is 12 when (3) is applied. SFDR can also be measured in Figure 19 as 72.28 dB, and (2) can be used to obtain the effective number of bits, which is 11, which suggests a 1-bit reduction in comparison with the SNR measurement.

#### 4.2.3. Transfer Function

Figure 19 shows the transfer function of the ADC, that is, the output with respect to its input. From this figure, we can conclude that the system works linearly.

#### 4.2.4. Frequency Sweep

**Figure 20:**
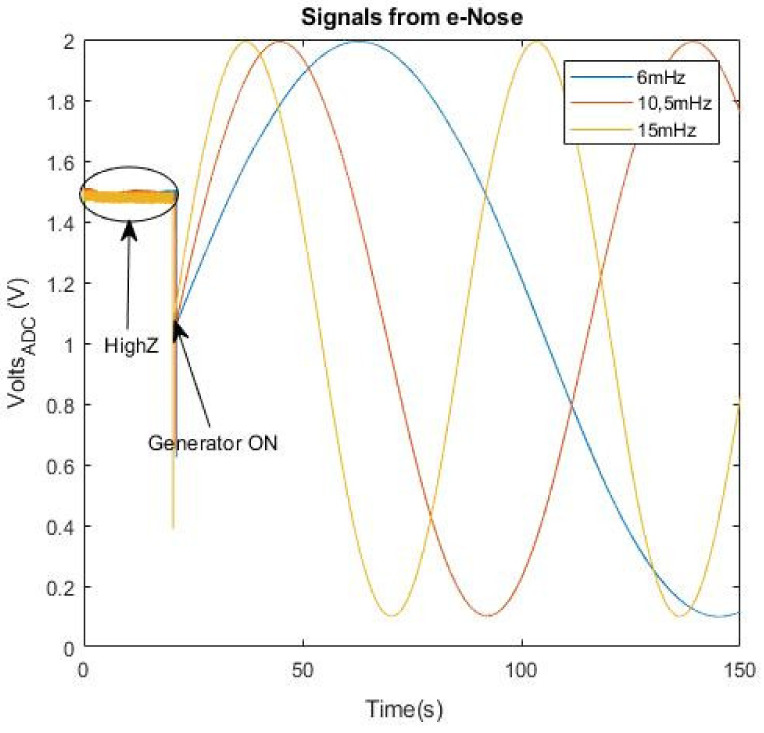
Signals for 6 mHz, 10.5 mHz and 15 mHz.

The implemented ADC was also tested with sine input signals of different frequencies that emulated the slope of the e-Nose signals showed in Figure 3 and Figure 4 (from 6 mHz to 15 mHz). The signals captured using the test in Figure 6 (real measurement) can be seen in Figure 20. The results of the test were identical to the ones showed above.

**Figure 21:**
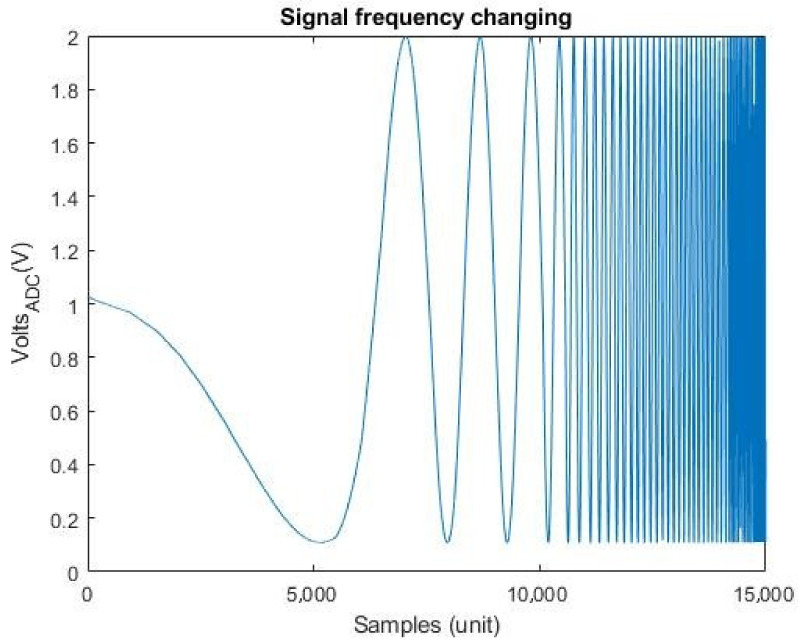
Sweep of a signal 1 mHz to 3 Hz.

Finally, Figure 21 shows the ADC output capture for an input signal with a sweep from 1 mHz to 3 Hz (the generator scroll was used to get the input signal). This figure shows the measured results obtained using the test shown in Figure 6.

## 5. Discussion

In this section, we compare the implementation results of our proposed ADC with [19,20], which propose ADCs taking advantage of the LDVS inputs of an FPGA. Table 3 shows the resolution, the ADC type, the resources on the FPGA used in each case and the type of application (low or high frequency). An extra column has been added to show the total number of four-input LUTs required by each design. It should be taken into account that this comparison is not fair because all designs were not implemented using the same device. As explained in Section 2.1, our application requires a minimum of 9 bits of resolution, so the 8-bit SAR proposed in [19] is not adequate even though it only needs 135 LUTs. Among the rest of the proposals, our design requires fewer four-input LUTs and meets our application’s resolution requirement.

It is worth mentioning that the work presented in this paper has added value because an SoC system was used and this allowed us to visualize the captured signal in real time, as shown in Figure 6, without using a PC. Additionally, this fact allows the implementation of a complete e-Nose classifier in the same SoC (acquisition, treatment, machine learning and classification).

## 6. Conclusions

In this work, an ADC converter to digitalize signals from an e-Nose with a gas sensor is proposed. It reached a resolution of 11 bits, which is enough for our application, with fewer resources than similar proposals, because it takes advantage of the signal characteristics: non-periodic, positive and with smooth changes. The designed ADC has an SNR of 75.97 dB, a SFDR of 72.28 dB and linear transfer function VADC/Vin. This proposal was verified for one gas sensor, but it is scalable for an array of sensors. This can be achieved by implementing up to 13 ADCs in the same FPGA working in parallel (there are 40 inputs in the FPGA card that can be configured as LVDS, and three inputs per ADC are required). If more than 13 sensors are required, the output of several sensors can be multiplexed. As the DE1-SoC card allows working with frequencies of 50 MHz, each ADC can work with 50 MHz/6553600 Hz = 7 MOS gas sensors multiplexed in time.

After including the required ADCs for the sensor array, the classification of samples could be carried out by implementing neuronal networks in the same FPGA, which optimizes the use of resources because it avoids the use of a PC or a similar device.

## Figures and Tables

**Table 1:** Specifications FPGA DE1-SoC.

FPGA Device
Cyclone V SoC 5CSEMA5F31C6 Device Dual-core ARM Cortex-A9 (HPS) 85 k Programmable Logic Elements 4450kbits embedded memory 6 Fractional PLLs 2 Hard Memory Controllers Fclk max 50 MHz

**Table 2:** Simulated signal acquisitions and ENOB results.

f	f_L_	N (Bits)	f_H_ (Hz)	R (Ω)	C (F)	ENOB (Bits)
35.5 mHz	100 Hz	16	6,553,600	10.61 × 10^3^	150 × 10^−9^	14.75

**Table 3:** Comparison of the implementation results of our proposed ADC with [19,20].

	Type and Bits	Resources (Device)	4-Input LUTs	Application
[19]	8-bit SAR	135 LUTs (Lattice XP2-17)	135	Low frequency
[19]	10-bit sigma delta	1000 LUTs (Lattice XP2-17) + 2 sysDSP blocks	1000	High frequency
[20]	12-bit sigma delta	700 LE (Stratix EP1S25)	700	High frequency
Our proposal	11-bit sigma delta	172 ALMs (Cyclone V)	344	Low frequency

## Data Availability

The following are available online at https://youtu.be/k0M14nm-XKw, 389 Video S1: Real time acquisition.
